# Comparison of the Dual Therapy of Ilaprazole-Amoxicillin and the Bismuth Quadruple Therapy of Ilaprazole-Amoxicillin-Furazolidone-Bismuth Glycyrrhizinate for Eradication of *Helicobacter pylori*

**DOI:** 10.3389/fphar.2022.771876

**Published:** 2022-04-27

**Authors:** Min Niu, Yan Zhou, Yunqian Xie, Xue Li, Yonggang Tian, Li Yao, Ximei Li, Hengjun Gao, Feihu Bai

**Affiliations:** ^1^ Department of Gastroenterology, People’s Hospital of Ningxia Hui Autonomous Region, Yinchuan, China; ^2^ School of Clinical Medicine, Ningxia Medical University, Yinchuan, China; ^3^ Department of Gastroenterology, The Second Affiliated Hospital of Hai Nan Medical College, Haikou, China; ^4^ Tongji Hospital, Institute of Digestive Disease, School of Medicine, Tongji University, Shanghai, China; ^5^ China Center for Helicobacter Pylori Molecular Medicine, Shanghai, China

**Keywords:** *Helicobacter pylori*, bismuth quadruple therapy, double therapy, clinical trial, eradication rate

## Abstract

**Objective:** The present study aims to compare the safety and efficacy of an amoxicillin/ilaprazole regimen with a bismuth quadruple regimen as the first-line treatment for eradicating *Helicobacter pylori* (*H. pylori*) infection.

**Methods:** This was an open-label, randomized, single-center study involving 450 patients with untreated *H. pylori* infection who were randomly assigned to an Ilaprazole-amoxicillin-furazolidone-bismuth glycyrrhizinate (IAFB) quadruple therapy group for 14 days, a bismuth quadruple therapy group for 10 days, or Ilaprazole-amoxicillin (IA) dual therapy group for 14 days. The 13C urea breath test determined that *H. pylori* had been eliminated 4–6 weeks after treatment. For patients who failed the first treatment, mucosal tissues (two gastric antrum and one gastric body) were taken under gastroscope for the culture of *H. pylori*, drug sensitivity, the CYP2C19 gene, and globular degeneration.

**Results:** In the intention-to-treat analysis, the eradication rates of *H. pylori* in the IAFB-14-day group, the IAFB-10-day group, and the IA-14-day group were 84.0, 79.3, and 88.0%, respectively. In the per-protocol analysis, the eradication rates in the three groups were 94.7, 87.5, and 93.0%, respectively. The resistance rates of patients who failed *H. pylori* eradication were 68.9% (22/32) for amoxicillin, 90.6% (29/32) for clarithromycin, 68.9% (22/32) for metronidazole, and 87.5% (28/32) for levofloxacin, and the extensive metabolizers of CYP2C19 polymorphism were 59.3% (19/32), the intermediate metabolizers were 34.4% (11/32), and the poor metabolizers were 6.3% (2/32).

**Conclusion:** For newly treated patients with *H. pylori* infection in China, the efficacy of IA therapy for 14 days was similar to IAFB quadruple therapy for 10 or 14 days with better compliance and less cost. Therefore, these therapies can be considered first-line regimens for empirical treatment.

**Clinical Trial Registration:** [http://www.chictr.org.cn/searchproj.aspx], identifier [ChiCTR2100052308].

## Introduction

*Helicobacter pylori* (*H. pylori*) is closely related to chronic gastritis, peptic ulcer, gastric cancer, gastric mucosa-associated lymphoid tissue lymphoma, and other digestive system diseases. Eradication of *H. pylori* is the most effective treatment. With the increasing resistance rate of clarithromycin and quinolones, the eradication rate of traditional triple therapy is only 80% (Graham and Fischbach, 2010), which is far from meeting the clinical needs. In recent years, quadruple therapy has been recommended as the first choice for *H. pylori* eradication, and the recommended course of treatment is 10 or 14 days (Malfertheiner et al., 2017). According to the Management of *H. pylori* infection-the Maastricht V/Florence Consensus Report and the ACG Clinical Guideline of Treatment of *Helicobacter pylori* Infection, dual therapy can be used for remedial treatment (Malfertheiner et al., 2017; Chey et al., 2018).

A number of studies have found that increasing the dose and frequency of amoxicillin and proton pump inhibitors (PPI) can improve the efficacy of an amoxicillin/PPI dual regimen (Sugimoto et al., 2012; Graham and Dore, 2016). First, some studies found that *H. pylori* entered the active replication state when pH > 6, and the pathogen was highly sensitive to amoxicillin (Labenz, 2001). Therefore, the use of PPI to inhibit gastric acid fully and continuously is conducive to the successful eradication of *H. pylori*. Second, amoxicillin is pH-dependent, which suggests that amoxicillin has higher stability and antibacterial effect with an increase in pH value (Sugimoto et al., 2012). Third, the effect of amoxicillin is time-dependent because it is absorbed rapidly, enters the plasma circulation, and excretes through the urine in a short time, and the bactericidal effect of multiple administrations is better (Graham and Dore, 2016). Therefore, a high dose of amoxicillin can improve the curative effect. The Maastricht-V Consensus and the *H. pylori* treatment consensus published by the American Gastroenterological Society in 2017 recommend that PPI be administered twice a day, which can maintain the gastric acid suppression effect and is conducive to eradication (Malfertheiner et al., 2017; Chey et al., 2018). Sugimoto et al. (2012) studied the correlation between the administration frequency of PPI and the effect of gastric acid inhibition. Their study found that the 24 h mean intragastric pH was associated with the frequency of administration of 40 mg of rabeprazole: intragastric pH 4.8 once daily, 5.7 twice a day, and 6.0 after four doses of rabeprazole. In this study, the gastric acid inhibition effect can be maintained by administration twice a day.

Therefore, this study aimed to investigate the eradication rate of *H. pylori* with dual therapy and bismuth quadruple therapy for 10 or 14 days. In this study, Ilaprazole was used as a PPI. Ilaprazole is a classic generation of PPI, which can effectively, stably, and lastingly inhibit gastric acid secretion and is not affected by CYP2C19 gene polymorphism. It has been used to treat acid-related diseases. In selecting antibiotics, the three antibiotics recommended by the Maastricht V/Florence Consensus Report and ACG Clinical Guideline (Malfertheiner et al., 2017; Chey et al., 2018), clarithromycin, metronidazole, and levofloxacin, have a high resistance rate and a low clinical cure rate. On the contrary, the resistance rate of the other three antibacterial drugs for eradicating *H. pylori*, amoxicillin (0–5%), tetracycline (0–5%), and furazolidone (0–1%), are still low (Liu et al., 2018). Regarding treatment choices, the guidelines point out that the recommended course of empirical treatment is 10 or 14 days. If the 10-day course of treatment can achieve a local eradication rate close or equal to 90%, then the 10-day treatment plan can be used as the first-line treatment option (Malfertheiner et al., 2017; Liu et al., 2018). In this experiment, the course of treatment for eradicating *H. pylori* was 10 or 14 days. The safety and effectiveness of the three regimens, as the first-line treatment for the Ningxia area *H. pylori* infection patients, provide high-efficiency treatment programs, improve the eradication rate in Ningxia, and control the source of infection, which is of great significance for the prevention and treatment of gastric-related diseases and gastric cancer.

## Methods

The study was approved by the ethics committee and the ethics committee approval document (LW2021038).

### Inclusion Criteria

Patients with a positive test for *H. pylori* infection detected by the C13 urea breath test (13C-UBT), aged between 14 and 70 years old, with no *H. pylori* eradication treatment in the past, and good compliance.

### Exclusion criteria

Patients that had taken drugs that affected the test results within 4 weeks, such as PPI, bismuth agents, antibiotics, and H2-receptor blockers; patients diagnosed with digestive tract tumors; patients who were pregnant or lactating; any drug allergy or intolerance.

### Calculation of Sample Size

The sample size was estimated based on the reported eradication rate of bismuth quadruple therapy (Sugimoto et al., 2012; Malfertheiner et al., 2017); the average *H. pylori* eradication rate is 90%. Assuming that the eradication rate of bismuth quadruple therapy in this trial is 90%, the non-inferiority threshold δ = −0.1 (−10%), α = 0.025 (unilateral), 1–β = 0.8, *p* = 90%, and a one-sided 97.5% confidence interval (CI). Using the SAS Version 9.2 software (SAS Institute Inc., Cary, NC), it was calculated that at least 104 cases of each group were required to show the non-inferiority of the two groups in the eradication rate of *H. pylori*. This was calculated based on the dropout rate not exceeding 20%, *n* = 104/0.8 ≈ 130. The sample size of each group would be 130 cases, with three groups total, so at least 390 qualified subjects needed to be included.

Using a digital sequence code randomly generated by a computer, all random number sequences were placed in a sealed opaque envelope. The patient randomly extracted a number. The researcher informed the patient of the group and the type, dosage, and time of the drugs taken, and the patient was admitted to the group to receive the corresponding treatment schedule. This research process was an open trial, and the researchers who specialize in *H. pylori*, as well as the outpatients and patients, were all aware of the treatment options.

The patients were randomly divided into three groups:

1) The IAFB-14-day group: 5 mg of Ilaprazole twice a day, 220 mg of bismuth glycyrrhizinate twice a day, 1.0 g of amoxicillin twice a day, and 0.1 g of furazolidone twice a day for 14 days.
2) The IAFB-10-day group: 5 mg of Ilaprazole twice a day, 220 mg of bismuth glycyrrhizinate twice a day, 1.0 g of amoxicillin twice a day, and 0.1 g of furazolidone twice a day for 10 days.
3) The IA-14-day group: 5 mg of Ilaprazole twice a day and 0.75 g of amoxicillin four times a day for 14 days.

During the treatment, the patients were given medication guidance, and a telephone follow-up was conducted on day nine or ten to understand the patients’ experience of the medication.

### Evaluation of the Effect of the Treatment

Compliance was evaluated 1–3 days after the treatment, and the number of tablets taken was counted to evaluate compliance. Compliance (%) = (the number of distributed tablets—the number of remaining tablets)/(the number of tablets to be taken) × 100%, and compliance (%) ≤80% were judged to be poor.

On the 30 days post-treatment, the patient was informed to come to the hospital to review 13C-UBT. If the result was negative, the eradication of *H. pylori* was successful. Eradication rate (%) = 13C-UBT negative cases/*H. pylori* eradication treatment (compliance ≥80%) and study of demographic and clinical characteristics on eradication rates in patients with ≥80% compliance.

Drug costs for the three treatment options were calculated based on the prevailing price standards of the Ningxia Hui Autonomous Region People’s Hospital (based on the Yinchuan 2019 drug pricing catalog).

### Culture, Drug Sensitivity, and Gene Analysis

For the patients who failed the first treatment, it was recommended that three samples of gastric mucosa should be taken after gastroscopy, including one sample of the gastric antrum and two samples of the gastric body. The three samples were collected and placed in the *H. pylori* isolation and preservation tube containing brain heart infusion. The bacterial culture, drug sensitivity test, and CYP2C19 gene polymorphism of *H. pylori* were analyzed to detect the drug sensitivity of *H. pylori-*positive strains to clarithromycin, amoxicillin, levofloxacin, furazolidone, tetracycline, and metronidazole. The disk diffusion method was used for the drug sensitivity test. According to the size of the inhibition zone (different antibiotics have different standards for the size of the inhibition zone), each sample was assessed for sensitivity, drug resistance, or mediation. A second sample of gastric antrum was collected and placed in an Eppendorf tube containing 1.5 ml of formalin to detect spherical deformation. This sample was stored in an ice bag and express shipped to the Shanghai Xinchao company for further testing.

Phenotypic resistance was determined by the Kirby–Bauer (K–B) disk-diffusion method. Suspensions of *H. pylori* strains were prepared to a final turbidity of 0.5 McFarland standard, then transferred to a new culture plate and cultured for 48 h. The antibiotic disks were used as follows: clarithromycin (15 μg), levofloxacin (5 μg), amoxicillin (10 μg), furazolidone (100 μg), tetracycline (30 μg), metronidazole (5 μg). The antibiotic disks were pressed on the agar surface of each plate and incubated for 48 h. The diameters of the inhibition rings were measured, and strains were identified as sensitive, intermediate, or resistant, according to the criteria.

DNA of *H. pylori* strains was extracted from a liquid transport medium by a HiPure Bacterial DNA kit (Magen BioSciences, Guangzhou, China). A polymerase chain reaction (PCR) was performed to amplify the genes 23S rRNA, gyrA, PBP1A, porD, oorD, 16S rRNA, and rdxA, which have been previously reported to be associated with antibiotic resistance. The reaction conditions of PCR were as follows: predenaturation at 94°C for 5 min, followed by 40 cycles of denaturation at 94°C for 10 s, annealing at 55°C for 20 s, extension at 72°C for 50 s each, and a final extension at 72°C for 5 min. PCR products were sequenced by Sanger sequencing. Several point mutations and amino mutations that have been previously reported to be associated with antibiotic resistance before were interpreted using Chromas 2.6.5 software. The CYP2C19*3 and CYP2C19*2 mutation sites of each sample and the type of metabolism were analyzed.

### Statistical Methods

SPSS statistical software 26.0 was used to statistically analyze the input data. Normally distributed measures were expressed as means ± standard deviation ( x ± s). A one-way ANOVA test was used for the comparison between groups. Count data were expressed as percentages. The chi-square test was used for comparison between groups. Treatment outcomes were analyzed using the intention-to-treat (ITT) and adherence-to-study protocol (per protocol, PP) analyses. *p* < .05 differences were considered statistically significant. Relevant influences were analyzed using the diagnostic odds ratio (OR) value. Differences were considered statistical when the upper 95% CI was less than one or the lower limit was greater than one.

## Results

**FIGURE 1:**
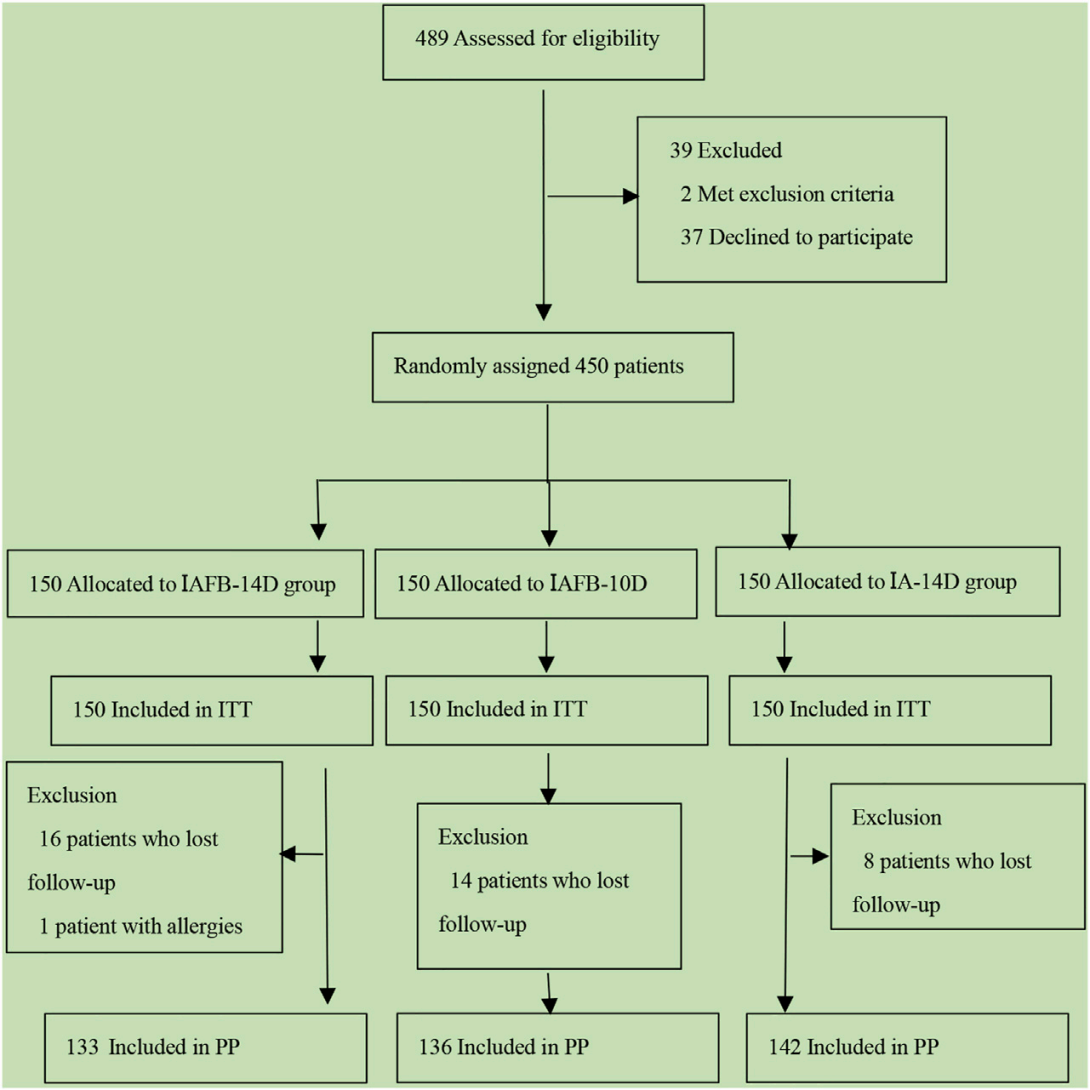
Flowchart of this study.

This study was a prospective, single-center, open-label, randomized, controlled clinical trial. Between August 1, 2019, and June 1, 2020, 450 patients with an *H. pylori* infection and 39 patients who were lost to follow-up at the Ningxia Hui Autonomous Region People’s Hospital were included in this study. The design of this study is outlined in a flow chart (Figure 1). The patients were randomly assigned to the IAFB-14-day group, the IAFB-10-day group, or the IA-14-day group according to the ratio of 1:1:1. In the IAFB-14-day group, 16 patients did not undergo the 13C-UBT after 4–6 weeks of treatment, and one patient stopped taking the drugs because of a rash after 2 days of treatment. Of the patients in the study, 14 patients in the IAFB-10-day group and eight patients in the IA-14-day group did not undergo the 13C-UBT after 4–6 weeks of treatment.

### Demographic and Clinical Characteristics

There was no significant difference in the baseline demographic and clinical characteristics (see Table 1), including gender, ethnicity, smoking history, drinking history, the sharing of tableware, history of chronic gastritis, ulcer history, and family history of gastric cancer between the two groups (*p* > .05). There was a significant difference in the age (44.10 ± 13.90 in the IAFB-14-day group, 44.25 ± 12.67 in the IAFB-10-day group, and 47.56 ± 13.04 in the IA-14-day group) and sharing of cups (with 16.5% (22/133) in the IAFB-14-day group, 12.5% (17/136) in the IAFB-10-day group, and 30.3% (43/142) in the IA-14-day group) between the three groups (*p* < .05).

**TABLE 1:** Demographic and clinical data of patients.

	IAFB-14days	IAFB-10days	IA-14days	*p*
Gender (male/female)	56/77	62/74	58/84	0.712
Age [(mean, SD), y]	44.10 ± 13.90	44.25 ± 12.67	47.56 ± 13.04	<0.05
Nation				
Han	122	127	129	0.733
Others	11	9	13	
Smoking	13	20	24	0.219
Regular alcohol use	14	11	22	0.141
Sharing tableware	87	101	95	0.241
Sharing cup	22	17	43	<0.05
History of gastroscopy				
Gastritis	60	58	42	0.017
Ulcer	20	14	15	0.402
Family history of gastric cancer	10	10	8	0.787

Note: Data are *n* (%), or mean (SD, standard deviation).

IA, Ilaprazole and amoxicilin.

IAFB, ilaprazole, amoxicillin, Furazolidone and bismuth.

^*^Compliance, taken >80% of tablets.

### *H. pylori* Eradication Rate

In the ITT analysis, the eradication rates of *H. pylori* in the IAFB-14-day, IAFB-10-day, and IA-14-day groups were 84.0% (126/150; 95% CI: 78.1–89.9%), 79.3% (119/150; 95% CI: 72.8–85.9%), and 88.0% (132/150; 95% CI: 82.7–93.3%), respectively. In the PP analysis, the eradication rates of *H. pylori* in the IAFB-14-day, IAFB-10-day, and IA-14-day groups were 94.7% (126/133; 95% CI: 90.9–98.6%), 87.5% (119/136; 95% CI: 81.9–93.1%), and 93.0% (132/142; 95% CI: 88.7–97.2%), respectively. There was no significant difference in the eradication rate between the three groups (ITT analysis: *p* = .125, PP analysis: *p* = .079; Table 2).

**TABLE 2:** Eradication rates in each regimen.

	IAFB-14days	IAFB-10days	IA-14days	*p*
ITT				
Eradication rate	84.0	79.3	88.0	0.125
95% CI	78.1–89.9	72.8–85.9	82.7–93.3	
PP				
Eradication rate	94.7	87.5	93.0	0.079
95% CI	90.9–98.6	81.9–93.1	88.7–97.2	

CI, confidence interval; ITT, intention-to-treat; PP, per-protocol.

### Adverse Reactions and Compliance

Adverse reaction grades mainly included mild (refers to a mild reaction or disease with no development of symptoms that generally does not require treatment), moderate (refers to obvious adverse reaction symptoms and moderate damage to vital organs or system functions), or severe (refers to serious damage of vital organs or system functions, resulting in disability or life-shortening or life-threatening). Adverse reactions included nausea, vomiting, abdominal pain, abdominal distension, diarrhea, dizziness, rash, tongue discoloration, and melena. All the adverse reactions disappeared after treatment. In the IAFB-14-day group, 31 patients had adverse reactions, including four with nausea, 11 with abdominal pain, nine with abdominal distension, six with diarrhea, and two with other symptoms (one with dizziness and one with a rash). There were 28 cases of adverse reactions in the IAFB-10-day group, including two cases of nausea, one of vomiting, four of abdominal pain, five of abdominal distension, and seven of diarrhea. There were 12 adverse reactions in the IA-14-day group, including one case of nausea, six of abdominal pain, four of abdominal distension, and one of diarrhea. One of the subjects in the IAFB-14-day group stopped taking the drugs because of a rash (medication possession ratio <80%). The incidence of adverse reactions was 8.5% (12/142) in the EA-14-day group, which was significantly lower than the 23.3% (31/133) and 20.1% (28/136) in the IAFB-14-day and IAFB-10-day groups, respectively (*p* < .05). There was no significant difference in compliance between the IAFB-14-day, IAFB-10-day, and IA-14-day groups (88.7 vs. 90.7% vs. 94.7%; *p* = .171; Table 3).

**TABLE 3:** Variety of adverse events in each regimen.

Variables	IAFB-14days	IAFB-10days	IA-14days	*p*
Total, *n* (%)	31	28	12	<0.050
AE grade				
Mild	26	21	11	0.558
Moderate	5	6	1	
Severe	0	0	0	
Adverse events				
Nausea	4	2	1	0.746
Vomiting	0	1	0	0.459
Abdominal pain	11	4	2	<0.050
Bloating	9	5	8	0.483
Diarrhea	6	7	1	0.477
Skin rash	1	0	0	0.520
Compliance	88.7	90.7	94.7	0.171

Adverse events were assessed in the per-protocol (PP) population. Compliance was indicative of patients who took at least 80% of study drugs.

NA, not applicable.

### Drug Sensitivity, Genes, and Immunohistochemistry in Patients With Treatment Failure

*H. pylori* were successfully cultured in 16 of 34 patients with treatment failure (47.1%)*.* The resistance rates of *H. pylori* were 12.5% (2/16) for amoxicillin, 12.5% (2/16) for furazolidone, 0% (0/16) for tetracycline, 75.0% (12/16) for clarithromycin, 93.8% (15/16) for metronidazole, and 81.3% (13/16) for levofloxacin. The resistance rates of the antibiotics did not differ significantly between the groups. The extensive metabolizers of CYP2C19 polymorphism were 59.3% (19/32), the intermediate metabolizers were 34.4% (11/32), and the poor metabolizers were 6.3% (2/32). The resistance rates of CYP2C19 polymorphism did not differ significantly between the groups (Table 4).

**TABLE 4:** Drug sensitivity and immunohistochemistry in patients with treatment failure.

	IAFB-14days	IAFB-10days	IA-14days	*p*
CLA resistance	4	5	3	0.944
LEV resistance	4	6	3	0.905
AMO resistance	1	0	1	0.401
FR resistance	1	1	0	0.654
TE resistance	0	0	0	—
MTZ resistance	5	6	4	0.504
CYP2C19 polymorphism				
EM	4	11	4	0.554
IM	2	5	4	
PM	1	0	1	
Spherical transformation	2	1	0	0.125

### Impact of Demographic and Clinical Characteristics on the Eradication Rate

The factors influencing the success of the three regimens were analyzed in the PP population (Table 5). Gender, age, ethnicity, smoking history, drinking history, the sharing of tableware, chronic gastritis, ulcer history, and family history of gastric cancer had no effect on the efficacy of the three methods.

**TABLE 5:** Factors influencing cure rates in the PP population.

Cure rate in subgroups	IAFB-14days	IAFB-10days	IA-14days
Gender			
Male	54/56	53/62	54/58
Female	72/77	66/74	78/84
OR (95% CI)	1.031 (0.630–1.688)	0.958 (0.585–1.572)	1.003 (0.619–1.624)
Nation			
Han	117/122	110/127	120/129
others	9/11	9/9	12/13
OR (95% CI)	1.172 (0.469–2.912)	0.866 (0.332–2.259)	1.008 (0.442–2.295)
smoking			
yes	12/13	18/20	16/24
no	114/120	101/116	116/118
*p*	0.972 (0.426–2.218)	1.034 (0.518–2.062)	0.678 (0.343–1.342)
Regular alcohol use			
yes	13/14	11/11	20/22
no	113/119	108/125	112/120
OR	0.978 (0.440–2.171)	1.157 (0.482–2.275)	0.974 (0.504–1.881)
Sharing tableware			
yes	84/87	93/101	88/95
no	42/46	26/35	44/47
OR	1.057 (0.632–1.769)	1.240 (0.694–2.215)	0.969 (0.598–1.636)
Sharing cup			
yes	21/22	13/17	37/43
no	105/111	106/119	95/99
OR (95% CI)	1.009 (0.524–1.942)	0.858 (0.398–1.851)	0.897 (0.532–1.511)
History of gastroscopy			
gastritis			
yes	54/60	52/58	38/42
no	72/73	67/78	94/100
OR (95% CI)	0.916 (0.558–1.491)	1.044 (0.635–1.715)	0.963 (0.571–1.621)
ulcer			
yes	17/20	13/14	10/15
no	109/113	106/122	122/127
OR (95% CI)	0.881 (0.438–1.771)	1.069 (0.481–2.375)	0.694 (0.300–1.604)
Family history of gastric cancer			
yes	10/10	9/10	7/8
no	116/123	110/126	125/134
OR (95% CI)	1.060 (0.426–2.641)	1.031 (0.404–2.629)	0.938 (0.330–2.662)

### Drug Cost

The cost of treatment mainly included the registration, examination, and treatment fees. The subjects of the three groups were all patients from the *H. pylori* special disease clinic at the Ningxia Hui Autonomous Region People’s Hospital. The treatment processes and examinations were the same, but the difference was in the treatment costs, i.e., the cost of the drugs. The cost of the medications for the three treatment plans was calculated according to the standard pricing of the Ningxia Hui Autonomous Region People’s Hospital at the time of the study. The price was expressed in U.S. dollars (USD; $), and CNY(Chinese Yuan) was converted into USD according to the exchange rate of October 2019: 1.00 USD = 6.69 RMB. The cost of the drugs of the three groups was calculated and compared directly. For the IAFB-14-day group, the total cost was $67.56 (28 Ilaprazole tablets were $47.23, 28 bags of bismuth glycyrrhizinate powder were $14.36, 112 amoxicillin capsules were $4.48, and 56 furazolidone tablets were $1.49). For the IAFB-10-day group, the total cost was $48.26 (20 Ilaprazole tablets were $33.74, 20 bags of bismuth glycyrrhizinate powder were $10.26, 80 amoxicillin capsules were $3.20, and 40 furazolidone tablets were $1.06). For the IA-14-day group, the total cost was $53.95 (28 Ilaprazole tablets were $47.23 and 168 amoxicillin capsules were $6.72). The cost of the drugs for the IA-14-day and the IAFB-10-day groups was lower than the IAFB-14-day group.

## Discussion

Antibiotic resistance is the most critical factor leading to eradication failure in *H. pylori* treatment. Currently, six kinds of antibiotics are recommended for its eradication, among which clarithromycin, metronidazole, and levofloxacin have high resistance rates, which significantly affect the eradication rate. In contrast to the high resistance rates of the three antibiotics mentioned, the current resistance rates of amoxicillin, tetracycline, and furazolidone are still low (Su et al., 2013; Liu et al., 2018). In the course of treatment, an increase in bismuth can increase the eradication rate of drug-resistant strains by 30–40%. At present, the guidelines recommend that bismuth quadruple therapy should be considered first when choosing the initial empirical treatment plan, and this method is recommended for 10 or 14 days. If it can be confirmed that the eradication rate of some local methods is close or up to 90% after 10 days of treatment, this plan can still be selected for treatment (Fallone et al., 2016; Malfertheiner et al., 2017).

In recent years, several domestic and foreign studies have shown that the success rate of dual therapy is low, which means that it is not suitable for the eradication of *H. pylori* in empirical first-line treatment. A study in South Korea has demonstrated that the eradication rate of combined therapy using amoxicillin and dexlansoprazole was 52% (Yoon et al., 2017). In the study by Taraq, 13 patients were treated for *H. pylori* infection with amoxicillin and dexamethasone. The results showed that the success rate of treatment according to the plan and intention was 53.8% (Attumi and Graham, 2014). However, these studies had small sample sizes and used different PPI, dosage, and frequency of PPI and amoxicillin, and they also lacked antibiotic resistance and CYP2C19 detection data. CYP2C19 is involved in hydroxylation metabolism of PPI. CYP2C19 gene polymorphism brings complexity to the use of PPI and is an important factor affecting individual differences in PPI efficacy (Denisenko et al., 2018).

In the present study, a 14-day combination of 5 mg of Ilaprazole twice a day and 750 mg of amoxicillin four times a day for 14 days resulted in an eradication rate of *H. pylori* similar to that of bismuth. Some recently published meta-analyses on combination therapy also draw similar conclusion that the high-dose double group and quadruple group had the same compliance with the eradication rate of *H. pylori*, but the high-dose double group was safer than quadruple group (Gao et al., 2020; Yun et al., 2021). However, dual therapy regimens have been less studied in other countries (e.g., Europe or the United States) and acquired lower cure rate in Korea (Kwack et al., 2016), so further clinical studies are needed to determine whether they can be used in other areas.

At the same time, from the adverse reactions of different treatment regimens, the incidence of adverse reactions to the dual therapy was significantly lower than the bismuth quadruple therapy. The primary adverse reaction in the combined therapy group was abdominal distension, while the main adverse reactions in the bismuth quadruple therapy group were abdominal pain and diarrhea, which were related to bismuth (Mégraud and Lehours, 2007). The compliance of patients in the bismuth quadruple therapy group was lower than the dual group, which may be related to the variety of drugs, the complexity of the medication methods, and more adverse reactions. We also analyzed the results of the drug sensitivity culture, the gene, and the immunohistochemistry results of globular transformation in patients with treatment failure. The results showed that the drug resistance rates of clarithromycin, metronidazole, levofloxacin, and tetracycline were 75.0, 93.8, 81.3, and 0%, respectively. The drug resistance rate of amoxicillin and furazolidone was 12.5%. In the follow-up, we will continue to assess the drug sensitivity culture of *H. pylori* in patients with treatment failure to further improve the experimental results. Drug resistance, the CYP2C19 gene, and Immunohistochemistry were analyzed in the three groups. The results showed no statistical difference between the groups, which may be related to the small number of gastric mucosa samples taken under gastroscopy in this study; the sample size will continue to be increased in follow-up experiments.

The results of this study showed that the drug cost of dual therapy was lower than the 14-day bismuth quadruple therapy, and the cost per patient was about $13.61. For patients, this reduces the burden of treatment, increases the willingness to choose this regimen, and increases treatment compliance. Considering long-term treatment for the prevention and treatment of drug resistance in the future and economic benefits to patients, only amoxicillin is selected for the combined treatment because it dramatically improves the patient’s compliance, reduces the occurrence of adverse reactions, and reduces the economic cost to patients.

In addition, the secondary drug resistance rate of amoxicillin is low, and there is a wide range of retreatment options after failure. In treating patients with an initial infection of *H. pylori*, compared with bismuth quadruple therapy, the use of amoxicillin can effectively avoid multiple and large-scale drug resistance, which is of great significance for the future treatment of *H. pylori* and its retreatment after failure.

There were still some limitations to this study. First, we did not monitor the 24 h intragastric pH during the whole treatment process, so we were unable to evaluate whether the administration of 5 mg of Ilaprazole twice a day successfully inhibited gastric acid secretion and maintained a 24 h intragastric pH > 6.0. Second, the sample size was not large enough, which may have led to potential bias in the sampling selection. The age and history of cup/glass sharing in the dual therapy group were significantly higher than in the bismuth quadruple therapy group. Although there was no effect on the eradication results, there was still bias. Third, this study was a single-center study, and the subject population has certain limitations.

Before combination therapy can become a first-line treatment alternative, we need to further study and explore dual therapy. First, a multicenter study should be conducted to expand the sample size and reduce bias. Second, PPI with stronger efficacy and longer duration should be selected to achieve appropriate intragastric pH. Vonoprazan, a newly developed potassium competitive acid blocker, is mainly metabolized by the CYP3A4 pathway *in vivo*, which is not affected by CYP2C19 gene polymorphism, and it has a long half-life and long acid inhibition (Echizen, 2016; Jung et al., 2017; Graham and Dore, 2018). Therefore, vonoprazan could be tried instead of PPI to eradicate *H. pylori*. In the treatment of *H. pylori*, the traditional PPI was replaced in the dual therapy. Third, to determine the best dose, frequency, and course of treatment, each drug in the dual therapy should be varied to achieve the maximum efficacy and formulate an optimal plan. Fourth, the choice of PPI in the dual therapy can be customized according to different genotypes of CYP2C19 or other genes (Flores-Treviño et al., 2018).

## Conclusion

To sum up, when selecting empirical treatment options for patients with initial *H. pylori* infection, our findings suggest that Ilaprazole-based dual 14-day therapy and bismuth quadruple 10-day therapy have similar eradication rates compared with bismuth quadruple 14-day therapy, and all can be considered as first-line options for empirical treatment. However, Ilaprazole-based dual 14-day therapy takes a variety of drugs, is simple to administer, has fewer adverse effects, has lower drug costs, and has a broader range of remedial treatment options available after treatment failure. Therefore, Ilaprazole-based dual 14-day therapy should be given priority when choosing a first treatment regimen for H. pylori-positive patients. Bismuth-containing regimens should be given priority, as appropriate, for the first eradication treatment in patients who smoke.

## Data Availability

The data presented in the study are available from https://www.ncbi.nlm.nih.gov/bioproject/822853, accession number PRJNA822853.
